# Temporomandibular Disorders and Oral Features in Systemic Lupus Erythematosus Patients: An Observational Study of Symptoms and Signs

**DOI:** 10.7150/ijms.38914

**Published:** 2020-01-01

**Authors:** Vito Crincoli, Maria Grazia Piancino, Florenzo Iannone, Mariella Errede, Mariasevera Di Comite

**Affiliations:** 1 Department of Basic Medical Sciences, Neurosciences and Sensory Organs, “Aldo Moro” University of Bari, Italy.; 2 Department of Surgical Sciences, University of Turin, Italy.; 3 Department of Emergency and Organ Transplantation, “Aldo Moro” University of Bari, Italy.

**Keywords:** Systemic Lupus Erythematosus, oral features, temporomandibular disorders, RDC/TMD

## Abstract

**Aims**: Systemic Lupus Erythematosus (SLE) is a connective tissue disease characterized by a wide range of pleomorphic pictures, including mucocutaneous, renal, musculoskeletal and neurological symptoms. It involves oral tissues, with hyposalivation, tooth decay, gingivitis, angular cheilitis, ulcers and glossitis. Temporomandibular disorders represent a heterogeneous group of inflammatory or degenerative diseases of the stomatognatic system, with algic and/or dysfunctional clinical features involving temporomandibular joint (TMJ) and related masticatory muscles. The aim of this study was to investigate the prevalence of oral manifestations and temporomandibular disorders (TMD) in SLE patients (Lp) compared with a control group.

**Methods**: Fifty-five patients (9 men and 46 women) with diagnosed Lupus were recruited in the study group. A randomly selected group of 55 patients, matched by sex and age, served as control group. The examination for TMD symptoms and signs was based on the standardized Research Diagnostic Criteria for Temporomandibular Disorders (RDC/TMD) through a questionnaire and clinical examination.

**Results**: Lupus patients complained more frequently (95.8%) of oral and TMJ symptoms (dysgeusia, stomatodynia, masticatory muscle pain during function, neck and shoulder muscles pain and presence of tinnitus) but only xerostomia (χ^2^=4,1548 *p*=0,0415), temple headache (χ^2^=4,4542 *p*=0,035) and the sensation of a stuck jaw (Mid-p-test *p*=0,043) were significant. About signs, cheilitis (*p*=0,0284) oral ulcers (χ^2^=4,0104 *p*=0,045) and fissured tongue are significantly more frequent in study group. The salivary flow was significantly decreased in the study group respect to the control one (p<0.0001). As regard to the oral kinematics, restricted movements (RM) in protrusion and left lateral movement were significantly different between study group and controls. In particular, 85,2% of Lp showed limited protrusion versus 56,4% of controls (χ^2^= 10,91 *p*<0,001); 59,3% of Lp had also a limitation during left lateral movement versus 47,3% of controls (T=2,225 *p*=0,0282). About bruxism, only the indentations on the lateral edges of the tongue were found in Lp group (72,7%), with a significant difference respect to controls (χ^2^=7,37 *p*=0,007).

**Conclusions:** While masticatory muscles have an overlapping behavior in both groups, the findings collected show a more severe TMJ kinematic impairment in Lp than in controls, with protrusion and left lateral movements significantly different. In addition, a remarkable reduction of salivary flow has been detected in Lp compared to controls. In conclusion, this autoimmune disease seems to play a role in oral manifestations and TMJ disorders, causing an increase in orofacial pain and an altered chewing function.

## Introduction

Lupus Erythematosus (LE) is a connective tissue disease and can be classified into two major forms: the systemic (SLE) and cutaneous (CLE) one. The former is a chronic, multisystem, rheumatic disorder, present at any age, with an etiology still unknown. It has a wide range of pleomorphic pictures, including mucocutaneous, renal, musculoskeletal and neurological symptoms.

The latter is further divided into three subtypes: acute (ACLE), subacute (SCLE) and chronic (CCLE) 1. The discoid LE (DLE) is the most common variant of CCLE, characterized by erythematosus macules and plaques, follicular occlusion, desquamation, telangiectasia and atrophy. The two categories can occur together or separately 2.

Annual estimated incidence of SLE from 1970s to 2000s is about 1-10/100.000 and the prevalence is about 5.8-13/100.000. Cutaneous forms are considered 2-3 times more frequent than systemic one 3. Women between 15 and 40 years are more frequently affected than men, with a preponderance of 6 up 10:1 4,5. Although Juvenile SLE (JSLE) is rarer, children usually experience higher disease activity and a more aggressive course compared to adults 6.

The mortality rate associated with the condition is around 3-5 times higher than that observed in the general population and is mostly attributed to the chronic inflammatory processes 7.

Pathogenesis of LE is multifactorial, involving genetic and environmental triggers 5. About genetic factors, population studies reveal a relationship between the susceptibility to LE and human leukocyte antigens (HLA) class II and class III genes polymorphism. In patients with the expression of HLA DR2 and DR3, SLE autoantibodies are produced: (i) extractable nuclear antigens or ENA, such as anti-Ro, anti-Sm, anti-La, anti-nRNP; (ii) antinuclear antibodies or ANA, such as anti-dsDNA and anti-ssDNA. Moreover, patients with complement component C1q deficit have the highest risk to develop LE, because of a reduced clearance of apoptotic bodies.

Also sex hormones and a defective hypothalamus-pituitary-adrenal (HPA) axis influence the susceptibility and the progression of the disease. In fact, increased estrogenic and reduced androgenic hormonal activity has been demonstrated in SLE patients (Lp) of both sex. Moreover, HPA axis is involved in the stress system, by increasing glucocorticoids, which results essential for prevention of dysregulated autoimmune answer. Environmental factors include infectious agents, diet, toxins/drugs, physical/chemical agents, hair dyes and tobacco smoke 8.

Clinically the disease has a variable course and a relapsing-remitting trend 4. Several organs and systems are affected (muscles, skeleton, joints, lungs, kidneys, skin, blood vessels, nervous system), so the pathology is characterized by different manifestations. The most frequent and characteristic feature is the malar rash (“butterfly” rash), associated with arthritis, glomerulonephritis, psychosis and seizure, serosity, fever, fatigue, weight loss, anemia 9. Mucocutaneous involvement includes alopecia, discoid lesions, photosensitivity and oral lesions 4. These are red macules or plaques, often localized on the hard palate, leukoplakic plaques, typical of buccal mucosa, lichenoid lesions, and ulcers on lips.

Hyposalivation predispose patients with SLE to an increased risk of developing caries, gingivitis and periodontal disease, fungal infections, especially with Candida species, exfoliative or angular cheilitis and glossitis, with hyperemic and smooth tongue because of loss of papillae. A high prevalence of oral complaints such as dysphagia, dysgeusia, and glossodynia is also present, while trigeminal neuralgia is rare [10,11,12,13,14,15,16 17,18].

Temporomandibular disorders (TMD) is a generic term referred to clinical conditions involving the jaw muscles and temporomandibular joint (TMJ). Such disorders are related to stress, age, gender, malocclusion and other systemic factors. It is estimated that about one third of adults suffer from TMD symptoms (TMDs) 19,20,21.

TMJ can be affected in patients with SLE as they have changes in the condyles, including flattening, cortical erosions, osteophytes, sub cortical cysts, sclerosis and gradual decrease in joint space due to granulation. This involvement can be linked to disease activity, leading to a breakdown of the cartilage matrix and bone destruction. Myopathies, with reduced masticatory muscle strength and atrophy, may be part of the disease condition or associated with a long term use of corticosteroid therapy 22,23,24,25.

Nevertheless, the literature lacks studies about TMJ and masticatory muscles involvement in patients with SLE, so the relationship between this disease and the temporomandibular disorders is unclear. Given this background, the aim of this study was to investigate clinically, through signs and symptoms, the prevalence of oral manifestations and temporomandibular disorders in Lp on drug therapy compared with a control group (CG), thus giving a complete survey of facial involvement in course of LES. The null hypothesis in this research was that Lp presented no differences in clinical characteristics and functional disabilities compared to a control group.

## Materials and Methods

This observational study was conducted from January 2016 to February 2019 at the School of Dentistry and the Department of Rheumatology, University of Bari, Italy, in accordance with the provisions of the Declaration of Helsinki. Ethical approval and informed consent were obtained from each human subject.

Fifty-five patients (9 men and 46 women) with diagnosed Lupus (Lp) were recruited in the study group.

Inclusion criteria were: age>18 years and Caucasian ethnic origin. Exclusion criteria were: traumatic diseases, head, oral or neck neoplasia, past or present chemotherapy and radiotherapy, neurological disorders, maxillofacial treatments, past or present orthodontic treatment. Patients with other rheumatic diseases were excluded from the study. A control group (CG) of 55 subjects, matched by sex with the study group and with no immune disease history, was randomly chosen among those presenting at the Dental Clinic. Patients age ranged between 18 and 85 years, with a mean age of 44,44 (SD = 15,04) years in the Lp group and 46 years (SD = 13,49) in the CG.

TMD signs and symptoms were valued following the standardized Research Diagnostic Criteria for Temporomandibular Disorders (RDC/TMD) 19.

A single experienced practitioner assessed TMD and orofacial manifestations through an anamnestic questionnaire and a clinical examination.

### Patients history

#### Oral symptoms

Through a questionnaire, patients listed the presence/absence of the following disorders:

*(i)Xerostomia*: characterized by dry mouth and associated to discomfort especially when they eat, speak, swallow and wear dentures. Less stringy and foamy saliva is produced 12.

*(ii)Dysgeusia*: defined a distorted gustatory perception. Patients report that they perceive bitter, sour, or metallic flavours 13.

*(iii)Stomatodynia*: a burning sensation in the mouth, associated with hurtful sensation or pain, even though oral mucosa appears clinically normal 14,15.

#### TMD symptoms

*TMDs:* they include muscle pain, neck and upper shoulders stiffness, pain at masticatory muscles during mandibular functions, arthralgia (tenderness or pain in TMJ area), a feeling of locked jaw, headaches, especially at the temples 16,19. Dizziness, earache and tinnitus are other less common problems that these patients also complained 26,27. Patients described how much the disease was serious according their perception by using VAS scale (from slight tenderness to unbearable pain) and if they had a periodical or continuous symptomatology since the disease was diagnosed.

*Myofascial pain (MP*): while palpation does not elicit sensations of tenderness or pain in healthy muscles, ache may be provoked by compression of contract or inflamed muscles. The following masticatory muscles were palpated bilaterally: anterior, medial and posterior temporalis muscles, masseter muscle, medial pterygoid muscle, lateral pterygoid muscle with its superior and inferior head, digastric (anterior and posterior belly) muscle and mylohyoid muscles. Palpation was performed applying soft but firm pressure to the muscle mainly with the palmar surface of the thumb and of the index finger.

### Clinical examination

#### Lupus oral signs

*Oral ulcers*: presence of oval or roundish sores inside the mouth.

*Petechiae*: red or brown pinpoint lesions not blanching on pressure, localized more frequently on the hard and soft palate 28.

*Erythema:* reddish and inflamed area on buccal mucosa.

*Fissured tongue*: anatomical condition of the tongue surface, usually asymptomatic. Grooves are distributed across the dorsal surface of the tongue and can vary in size and depth.

*Cheilitis of lower lip:* it is an inflammation state, characterized by redness, swelling and ulcers on lower lip.

*Hyposalivation:* the test was conducted asking the patient to spit saliva accumulated in the floor of the mouth without stimulation in a graduated tube every 60 seconds. The collection period lasted 5 minutes 29.

Other oral characteristics analyzed were the integrity of the dental arches or presence of partial or total edentulism, the presence/absence of prostheses (mobile, fixed or both).

#### TMJ signs

*TMJ sounds (TMJs):* they were appreciated by palpation on each side separately on mandible movement. They can be classified in: (i) clicking, (ii) crepitation. Clicking is defined as a single, clear joint sound of short duration. Crepitation is a sound similar to a rough multiple, gravel-like sound 20.

*Bruxism (BRUX):* it is a stereotypical jaw movement, characterized by clenching and gnashing of the teeth, during sleep or when awake 30. By time, flattening of the dental cusps or dental mobility can occur. Bruxism is considered pathological when it causes myalgia (due to a prolonged vasoconstriction and to accumulation of catabolites in the muscle tissue) and joint pain 31.

*Opening derangement (OD):* in a healthy condition, the mandible-opening path (observing the lower midline) is straight. Alterations of the opening trajectory are:

(i) deviation: any shift of the jaw midline during opening that disappears with continued opening (a return to midline);

(ii) deflection: any shift of the midline to one side that increases with opening and persists at maximum opening 32.

#### Restricted movements (RM)

They are classified as:

(i) reduced opening: in a healthy system, the mouth opens by between 53 and 58 mm. Taking into account overbite 33, a restricted mandibular opening is considered to be any distance of <40 mm;

(ii) reduced right and left lateral excursions, measured from upper to lower midline, when the distance is <8 mm;

(iii) a mandibular advancement: it is considered reduced when <7mm 34,35.

### Statistical analysis

Continuous data were presented as mean and Standard Deviation (SD) and the comparisons between Lp and controls were assessed by means of Student's T test for unpaired samples. Categorical data were expressed as number and percentage and Chi-squared (with Mantel-Haenszel or Yates' corrections). Mid-P or Fisher Exact Tests were employed to compare two groups. A two-tailed *p* value ≤0.05 was considered as statistically significant. Statistical analyses were performed using Prism (GraphPad software, version 6.0, San Diego, California).

## Results

### Characteristics of Lupus patients (Lp) and controls

The prevalent form of disease was the systemic one (SLE), found in the 90,9% of Lp. The age at diagnosis varied between 12 and 84 years (mean=32,9 years, SD = 16,07) with a disease mean duration of 8,04 years (SD = 8); 43,6% of patients had the pathology for less than 5 years.

The two groups, matched for age and sex, resulted similar for sociodemographic aspects, except for educational degree (χ**^2^**=9,4184 *p*=0,0242) and occupation (χ**^2^**=21,6894 *p*=0.0014). About education, high school degree was prominent in both groups, but graduates were more than double among controls. As regard to occupation, among Lp group housewives and jobless prevailed, while among controls public employees were prominent (Table 1).

Clinical characteristics of Lp patients and controls are reported in Table 2. The principal drugs for Lp patients and controls are reported in Table 3.

### Lupus oral symptoms

Twenty-nine Lp (52,7%) complained of one or more oral symptoms compared to 24 of controls (43,6%). A statistically significant difference between two groups was found only for xerostomia (Table 4).

### TMD symptoms

The assessment of TMDs showed that 94,5% Lp and 90,9% of controls complained one or more symptoms. Statistically significant differences weren't found between the two whole groups (χ^2^=0,5392 *p*=0,463).

Arthralgia, temple headache, sensation of a stuck jaw, masticatory muscle pain during function, neck and shoulder muscles pain and presence of tinnitus are more frequent in Lp than in controls, but only temple headache (χ^2^=4,4542 *p*=0,035) and the difficulty in opening mouth (Mid-p-test *p*=0,043) are significant (Table 5).

Myofascial pain (MP) evoked by palpation was detected in 70,9% Lp and 76,4% of controls, revealing a similar frequency between Lp and controls (χ^2^=0,4215 *p*=0,516). Data collected for each muscle couple are reported in Table 6.

### Lupus oral signs

At the clinical examination, 52,7% of Lp have at least one oral sign respect to 43,6% of controls, but this difference is not statistically significant (χ^2^=0.91 *p=*0,340). However, there are statistically significant differences between the two groups for some observed signs. Cheilitis, oral ulcers and fissured tongue are more frequent in study group. Other signs (oral respiration, gingival recessions, herpes labialis, BMS) are more frequently observed in controls than Lp (χ^2^=4,6383 *p=*0,031). Table 7 lists the main findings collected from the oral examination.

**Figure 1:**
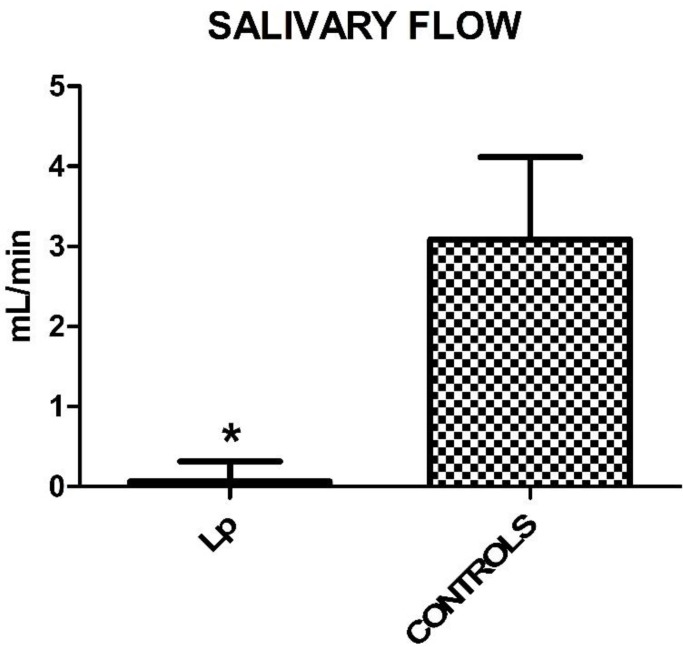
Mean values ± SD of Lp and control group. Lp patients show a reduced salivary flow.

Regarding the extent of salivary flow, measured in 5 minutes, there was a statistically significant reduction in the Lp compared to controls (Student's T Test, *p*<0,0001). Mean values and SD are: 0.254 ± 0.395 for Lp and 3.197 ± 0.571 for control group (Figure 1).

### TMJ signs

As regard to restricted movements (RM), protrusion and left lateral movement were significantly different between Lp and controls. In particular, 85,2% of Lp showed limited protrusion versus 56,4% of controls (χ^2^= 10,91 *p*<0,001); 59,3% of Lp had a limitation during left lateral movement versus 47,3% of controls (T=2,225 *p*=0,0282).

The prevalence of Opening derangement (OD) was higher in Lp (45,20%) than in controls (44,24%), although this difference was not statistically significant (χ^2^=0,05 *p*=0,5347). Measurement data related to movements are reported in Table 8.

Bruxism was more frequent in control group than Lupus one (38,2% versus 32,7%). No significant difference between the two groups was found for wear facets and oral frictional hyperkeratosis (*morsicatio buccarum*). Only the indentations on the lateral edges of the tongue were found in Lp group (72,7%), with a significant difference respect to controls (χ^2^=7,37 *p*=0,007). Table 9 lists parafunction (bruxism and clenching) signs.

Finally, joint sounds were more frequent in controls (25,5%) than Lp (16,4%) but with no statistically significant difference.

## Discussion

The present study analyzed the prevalence of symptoms and sign of both mucosal damage and temporomandibular dysfunction in Lp compared with healthy controls. The present cohort is larger respect to the sample size of other studies investigating TMD and LE: 55 Lp versus 2 in Liebling and Gold 36, 2 in Simoes et al. 37, 22 in Aliko et al. 23, 25 in Aceves-Avila et al. 38, 37 in Jonsson et al. 39. Lopez-Labady's work examined 90 Lp, but only oral manifestations were described, while TMD were omitted 1.

About sociodemographic characteristics, women (46 females and 9 males) are more affected than men, in accordance with the literature 4,5. Jobless and housewives were most represented in Lp, while public employees were most represented among controls. About education, graduates were more than double among controls. These data could be explained with a progressive physical disability in patients with SLE. In fact, several comorbidities (Table 2), such as renal (χ**^2^**=31,4789 p<0,001), lung (χ**^2^**=13,8318 p<0,001) and blood diseases (χ**^2^**=28,1928 p<0,001), lead to an impairment of patients' quality of life.

Mucosal symptoms and signs were evaluated in Lp and control group: xerostomia, among symptoms, and cheilitis, fissured tongue and ulcers, among signs, show statistically significant findings. In detail, 30,9% of Lp versus 14,5% of controls (χ**^2^** =4,1548 *p*=0,0415) complained xerostomia, with a feeling of mouth dryness (sicca syndrome). The main cause appears to be a lymphocytic sialadenitis associated with a secondary Sjögren's syndrome. In fact, according to Brennan et al., 7,5-30% of Lp have Sjogren syndrome, with cheilitis, erythema, hyperkeratotic areas, ulcerations, periodontal diseases, atrophic or fissured tongue, caries, candidiasis 4,10,29,40,41. Also drugs can cause xerostomia: 74,5% of Lp take corticosteroids (*p*=0,001) from the diagnosis of the disease, often in addition to others drugs, such as hydroxychloroquine (56,4%), Mycophenolate Mofetil (27,3%), azathioprine (14,5%), cyclosporine (10,9%). This remarkable symptom is confirmed by the salivary test, with a significantly reduced salivary flow in Lp patients. Consequently, the hyposalivation may be responsible of cheilitis (*p*=0,0284), fissured tongue (*p*=0,006) and oral ulcers (*p*=0,045), statistically more frequent in Lp than controls.

A higher prevalence of TMD symptoms was detected in Lp (94,5%) compared to the respective outcomes in healthy patients (90,9%). However, only temple headache (χ**^2^**=4,4542 *p*=0,035) and sensation of stuck jaw (Mid-p-test *p*=0,043) are significant (Table 5). Myopathies, with reduced muscle strength and atrophy, may be part of the disease condition or associated with the continuous use of corticosteroid therapy. Myofascial pain (MP) evoked by palpation was detected in 70,9% Lp and 76,4% of controls (χ^2^=0,4215 p=0,5162), so masticatory muscles show overlapping data in both groups (Table 6). The results of the present investigation are in accordance with literature. To our knowledge, in fact, in no previous examined study, pain was elicited during muscles palpation, except in Bade's study, who valuated only one patient with SLE (masseter, anterior temporal and lateral pterygoid muscles were painful) 42.

Also temporomandibular joint (TMJ) involvement can affect patients with LES, as they have changes in the condyles, including flattening, erosions, osteophytes and sclerosis. These alterations can be linked to disease activity, leading to loss of joint cartilage and bone destruction.

A valuated symptom was arthralgia (pain around TMJ area). It is more frequent in Lp (30,9%) respect to controls (18,2%), but it isn't statistically significant. This result is in contrast with previous studies, where pain during TMJ palpation was always found 23,39,42.

In this investigation, the presence of TMJ sounds, was overall more evident in controls (25,5%) than Lp (16,4%), but not significantly different. Also Aliko 23, and Jonsson 39 did not found a significant difference in Lp and controls.

Maybe the explanation is that crepitation often indicates structural damage to the TMJ and the drugs early given in LE could have the potential to reduce or slow down joint damage 22. Moreover, the "healthy" sample was selected from people attending at Dental Clinic, who could complain TMD more frequently than general population, and this could be a limitation of the present study.

The main expected finding was a more severe restriction on mandibular movements in Lp patients than in controls. Protrusion and left lateral movement were significantly different between the two groups. In particular, 85,2% of Lp showed limited protrusion versus 56,4% of controls (χ^2^= 10,91 *p*<0,001); 59,3% of Lp had a limitation during left lateral movement versus 47,3% of controls (T=2,225 *p*=0,0282).

However, the mouth opening value (mean=45,20 ± 8,615) was not statistically significant respect to control group (25,5% of Lp versus 23,6% of controls). Also Aliko showed a restricted opening only in 4,5% of patients 23, while for Jonsonn this sign is statistically significant 39.

## Conclusions

The aim of this observational study was to investigate the prevalence of TMD symptoms and signs as well as oral implications in patients with SLE. While masticatory muscles have an overlapping behavior in both groups, the findings collected show a more severe TMJ kinematic impairment in Lp patients than in controls, with protrusion and left lateral movements significantly different. Also a remarkable reduction of salivary flow was detected in Lp compared to controls (*p*<0,0001).

Lupus, like other autoimmune diseases, seems to play a role in oral and TMJ alterations, causing an increase in orofacial pain and an impairment of jaw mobility. An interdisciplinary collaboration between the stomatologist and the rheumatologist would be appropriate, thus giving a complete survey of facial involvement in course of LES and a more efficient treatment of this disease.

## Figures and Tables

**Table 1:** Sociodemographic characteristics of Lupus patients (Lp) and controls.

Sociodemographic characteristics	Lp	Controls	Test	*p* Value
**Age, mean±SD**	44,44±15,04	46±13,49		
**Sex, *n* (%)**				
Male	9 (16,4%)	9 (16,4%)		
Female	46 (83,6%)	46 (83,6%)		
**Educational degree, *n* (%)**			χ^2^=9,4184	0,0242
Primary	8 (14,5%)	5 (9,1%)		
Secondary	17 (30,9%)	7 (12,7%)		
High	25 (45,5%)	29 (52,7%)		
Academic	5 (9,1%)	14 (25,5%)		
**Occupation, *n* (%)**			χ^2^=21,6894	0,0014
Housewife	18 (32,7%)	13 (23,6%)		
Retired	5 (9,1%)	5 (9,1%)		
Office Worker	12 (21,8%)	11 (20%)		
Self Employed	3 (5,5%)	4 (7,3%)		
Not Employed	12 (21,8%)	1 (1,8%)		
Public Employee	2 (3,6%)	16 (29,1%)		
**Marital status, *n* (%)**			χ^2^=7,2039	0,0657
Married	29 (52,7%)	39 (70,9%)		
Widower	1 (1,8%)	2 (3,6%)		
Single	21 (38,2%)	14 (25,5%)		
Divorced	4 (7,3%)	0		

**Table 2:** Comorbidity in Lp and controls.

Concomitant diseases	Lp, *n* (%)	Controls *n* (%)	Test	*p* Value
Cardiopathy	9 (16,4%)	2 (3,6%)	χ^2^= 4,9045	0,027
Diabetes mellitus	2 (3,6%)	0	Fisher exact test	0,248
Esophageal disease	8 (14,5%)	3 (5,5%)	χ^2^= 2,5253	0,112
Gastritis	2 (3,6%)	0	Fisher exact test	0,248
Hypertension	12 (21,8%)	6 (10,9%)	χ^2^= 2,3913	0,122
Hypovitaminosis D	6 (10,9%)	0	Fisher exact test	0,016
Lung disease	14 (25,5%)	0	χ^2^= 13,8318	<0,001
Osteoporosis	10 (18,2%)	1 (1,8%)	χ^2^= 6,4646	0,011
Presence of removable prosthesis	4 (7,3%)	6 (10,9%)	χ^2^= 0,4400	0,507
Raynaud syndrome	10 (18,2%)	0	χ^2^= 8,9100	0,003
Thyroid disease	12 (21,8%)	5 (9,1%)	Mid-p-test	0,036
Renal disease	26 (47,3%)	0	χ^2^= 31,4789	<0,001
Blood disease	24 (43,6%)	0	χ^2^= 28,1928	<0,001
Neurological disease	7 (12,7%)	0	Fisher exact test	0,006

**Table 3:** Lp and controls' drugs.

Drugs	Lp, *n* (%)	Controls* n* (%)	Test	*p* Value
Azathioprine	8 (14,5%)	0	Fisher exact test	0,003
Belimumab	2 (3,6%)	0	Fisher exact test	0,248
Cyclophosphamide	3 (5,5%)	0	Fisher exact test	0,122
Cyclosporine	6 (10,9%)	0	Fisher exact test	0,0135
Corticosteroids	41 (74,5%)	0	Fisher exact test	<0,001
Hydroxychloroquine	31 (56,4%)	0	Fisher exact test	<0,001
Leflunomide	1 (1,8%)	0	Fisher exact test	0,500
Methotrexate	4 (7,3%)	0	Fisher exact test	0,059
Mycophenolate Mofetil	15 (27,3%)	0	Fisher exact test	<0,001
Rituximab	2 (3,6%)	0	Fisher exact test	0,248

**Table 4:** Subjective complaints of oral discomfort in Lp and controls.

Oral symptoms	Lp, *n* (%)	Controls, *n* (%)	χ^2^	*p V*alue
Xerostomia	17 (30,9%)	8 (14,5%)	4,1548	0,0415
Dysgeusia	5 (9,1%)	2 (3,6%)	1,3731	0,241
Stomatodynia	4 (7,3%)	3 (5,5%)	0,1526	0,696

**Table 5:** TMD symptoms in Lp and controls.

TMDs	Lp, *n* (%)	Controls, *n* (%)	Test	*p* Value
Arthralgia	17 (30,9%)	10 (18,2%)	χ^2^= 2,4052	0,121
Temple headache	29 (52,7%)	18 (32,7%)	χ^2^= 4,4542	0,035
Sensation of stuck jaw	13 (23,6%)	6 (10,9%)	Mid-p-test	0,043
Masticatory muscle pain during function	14 (25,5%)	8 (14,5%)	χ^2^= 2,0455	0,153
Neck and shoulder muscles pain	38 (69,1%)	35 (63,6%)	χ^2^= 0,3665	0,545
tinnitus	16 (29,1%)	10 (18,1%)	χ^2^= 1,8132	0,178

**Table 6:** Myofascial pain in Lp and controls.

Muscle	Lp, *n* (%)	Controls,* n* (%)	Test	*p* Value
Anterior temporalis muscles	21 (38,2%)	17 (30,9%)	χ^2^= 0,6433	0,423
Medial temporalis muscles	16 (29,1%)	12 (21,8%)	χ^2^= 0,7666	0,381
Posterior temporalis muscles	11 (20,0%)	10 (18,2%)	χ^2^= 0,0589	0,808
Superficial masseter muscles	29 (52,7%)	25 (45,5%)	χ^2^= 0,5820	0,446
Deep masseter muscles	29 (52,7%)	26 (47,3%)	χ^2^= 0,3273	0,567
medial pterygoid muscles	29 (52,7%)	30 (54,5%)	χ^2^= 0,0366	0,848
lateral pterygoid muscle	30 (54,5%)	37 (67,3%)	χ^2^= 1,8709	0,171
digastric muscle - anterior belly	10 (18,2%)	11 (20,0%)	χ^2^= 1,0589	0,808
digastric muscle - posterior belly	13 (23,6%)	18 (14,5%)	χ^2^= 1,4714	0,225
Mylohyoid muscles	14 (25,5%)	11 (20,0%)	χ^2^= 0,4659	0,495

**Table 7:** Oral signs for Lp and controls.

Oral signs	Lp, *n* (%)	Controls, *n* (%)	Test	*p* Value
Candidiasis	1 (1,8%)	0	Fisher exact test	0,500
Cheilitis	5 (9,1%)	0	Fisher exact test	0,0284
Erythema	3 (5,5%)	0	Fisher exact test	0,122
Petechiae	3 (5,5%)	0	Fisher exact test	0,122
Fissured tongue	7 (12,7%)	0	Fisher exact test	0,006
Oral ulcers	11 (20%)	3 (5,5%)	χ^2^= 4,0104	0,045
Erythematous and hyperkeratotic areas	2 (3,6%)	0	Fisher exact test	0,248
Others	4 (7,3%)	12 (21,8%)	χ^2^= 4,6383	0,031

**Table 8:** Restricted Movements (RM).

Measurements (mm); mean±SD	Lp	Controls	T Student	*p* Value
Opening	45,20 ±8,61	44,24 ± 7,65	0,6228	0,5347
Laterotrusion right	5,85 ± 3,49	6,86 ± 2,5	1,681	0,0957
Laterotrusion left	6,13 ± 4,02	7,636 ±2,98	2,225	0,0282
Protrusion	4,09 ± 2,36	6,091 ± 2,75	4,069	< 0,0001

**Table 9:** Parafunctional signs.

Parafunctional signs	Lp	Controls	Test	*p* Value
Wear facets	32 (58,2%)	25 (45,5%)	χ^2^= 1,7842	0,550
Oral frictional hyperkeratosis	26 (47,3%)	19 (34,5%)	χ^2^=1,8427	0,175
Indentations on lateral edges of tongue	40 (72,7%)	25 (45,5%)	χ^2^=7,3709	0,007

## Abbreviations

LE: Lupus Erythematosus
SLE: Systemic Lupus Erythematosus
CLE: Cutaneous Lupus Erythematosus
SCLE: Subacute Cutaneous Lupus Erythematosus
ACLE: Acute Cutaneous Lupus Erythematosus
CCLE: Chronic Cutaneous Lupus Erythematosus
DLE: Discoid Lupus Erythematosus
HLA: Human Leukocyte Antigens
ENA: Extractable Nuclear Antigens
ANA: Anti Nuclear Antigens
HPA: Hypothalamus- Pituitary- Adrenal
ANUG: Acute Necrotizing Ulcerative Gingivitis
Lp: Lupus Patients
CG: Control Group
TMJ: Temporomandibular Joint
TMD: Temporomandibular Disorders
SD: standard deviation
TMDs: Symptoms of Temporomandibular Disorders
MP: Myofascial Pain
TMJs: Sounds of Temporomandibular Joint
BRUX: bruxism
BMS: Burning Mouth Syndrome
RM: Restricted Movements
OD: Opening Derangement
RDC/TMD: Research Diagnostic Criteria for Temporomandibular Disorders
VAS: Visual Analogue Scale
